# Advanced MRI analysis to detect white matter brain injury in growth restricted newborn lambs

**DOI:** 10.1016/j.nicl.2019.101991

**Published:** 2019-08-23

**Authors:** Atul Malhotra, Tara Sepehrizadeh, Thijs Dhollander, David Wright, Margie Castillo-Melendez, Amy E. Sutherland, Yen Pham, Michael Ditchfield, Graeme R. Polglase, Michael de Veer, Graham Jenkin, Kerstin Pannek, Rosita Shishegar, Suzanne L. Miller

**Affiliations:** a Monash Newborn, Monash Children's Hospital, Melbourne, Australia; b Department of Paediatrics, Monash University, Melbourne, Australia; c The Ritchie Centre, Hudson Institute of Medical Research, Melbourne, Australia; d Monash Biomedical Imaging, Monash University, Melbourne, Australia; e The Florey Institute of Neuroscience and Mental Health, Melbourne, Australia; f The Florey Department of Neuroscience and Mental Health, University of Melbourne, Melbourne, Australia; g Department of Neuroscience, Central Clinical School, Monash University, Australia; h Department of Obstetrics and Gynaecology, Monash University, Melbourne, Australia; i Diagnostic Imaging, Monash Health, Melbourne, Australia; j Commonwealth Scientific and Industrial Research Organisation, Brisbane, Australia; k The Turner Institute for Brain and Mental Health, Monash University, Melbourne, Australia; l CSIRO Health and Biosecurity, Parkville, Victoria, Australia

**Keywords:** Fixel-based analysis, Tractography, Myelination, Structure, Fibre

## Abstract

**Background:**

Fetal growth restriction (FGR) is a serious pregnancy complication associated with increased risk of adverse neurodevelopment and neuromorbidity. Current imaging techniques, including conventional magnetic resonance imaging (MRI), are not sensitive enough to detect subtle structural abnormalities in the FGR brain. We examined whether advanced MRI analysis techniques have the capacity to detect brain injury (particularly white matter injury) caused by chronic hypoxia-induced fetal growth restriction in newborn preterm lambs.

**Methods:**

Surgery was undertaken in twin bearing pregnant ewes at 88–90 days gestation (term = 150 days) to induce FGR in one fetus. At 127 days gestation (~32 weeks human brain development), FGR and control (appropriate for gestational age, AGA) lambs were delivered by caesarean section, intubated and ventilated. Conventional and advanced brain imaging was conducted within the first two hours of life using a 3T MRI scanner. T1-weighted (T1w) and T2-weighted (T2w) structural imaging, magnetic resonance spectroscopy (MRS), and diffusion MRI (dMRI) data were acquired. Diffusion tensor imaging (DTI) modelling and analysis of dMRI data included the following regions of interest (ROIs): subcortical white matter, periventricular white matter, cerebellum, hippocampus, corpus callosum and thalamus. Fixel-based analysis of 3-tissue constrained spherical deconvolution (CSD) of the dMRI data was performed and compared between FGR and AGA lambs. Lambs were euthanised immediately after the scans and brain histology performed in the regions of interest to correlate with imaging.

**Results:**

FGR and AGA lamb (body weight, mean (SD): 2.2(0.5) vs. 3.3(0.3) kg, *p* = .002) MRI brain scans were analysed. There were no statistically significant differences observed between the groups in conventional T1w, T2w or MRS brain data. Mean, axial and radial diffusivity, and fractional anisotropy indices obtained from DTI modelling also did not show any statistically significant differences between groups in the ROIs. Fixel-based analysis of 3-tissue CSD, however, did reveal a decrease in fibre cross-section (FC, *p* < .05) but not in fibre density (FD) or combined fibre density and cross-section (FDC) in FGR vs. AGA lamb brains. The specific tracts that showed a decrease in FC were in the regions of the periventricular white matter, hippocampus and cerebellar white matter, and were supported by histological evidence of white matter hypomyelination and disorganisation in corresponding FGR lamb brain regions.

**Conclusions:**

The neuropathology associated with FGR in neonatal preterm lambs is subtle and imaging detection may require advanced MRI and tract-based analysis techniques. Fixel-based analysis of 3-tissue CSD demonstrates that the preterm neonatal FGR brain shows evidence of macrostructural (cross-sectional) deficits in white matter subsequent to altered antenatal development. These findings can inform analysis of similar brain pathology in neonatal infants.

## Highlights

• FGR brain injury can be subtle, and not easily detected on conventional imaging.
• Fixel-based analysis showed differences in fibre content of FGR lamb brain tracts.
• Histological stain confirmed myelination deficits in corresponding brain regions.

## 1 Introduction

Fetal growth restriction (FGR) is a serious pregnancy complication that can lead to significant perinatal compromise, neonatal morbidity and risk of long term adverse outcomes (Malhotra et al., 2019). Placental insufficiency is the principal cause of FGR, leading to progressive restriction of oxygen and nutrient delivery to the developing fetus which, in turn, is linked with impairments in brain structure and function (Miller et al., 2016). White matter injury is the most common neuropathology responsible for the adverse neurological and neurodevelopmental outcomes of FGR infants (Tolcos et al., 2011; Saunavaara et al., 2017; Tolcos et al., 2017), but neuropathology is generally multicellular, diffuse and often subtle (Miller et al., 2016). Adverse neurological outcomes depend on the age of onset of FGR, severity of growth restriction, degree of fetal cardiovascular adaptation (traditionally known as “brain sparing”) and gestation at birth (Malhotra et al., 2019).

The early and accurate detection of brain injury in the growth restricted fetus and newborn remains a significant challenge (Malhotra et al., 2017). Current diagnostic imaging tools are essentially limited to antenatal and more commonly postnatal cranial ultrasound and, in selected and severe cases, magnetic resonance imaging (MRI). However, clinical implementation of these techniques is subject to high variability, and generally poor sensitivity and specificity for detecting FGR related brain injury (Malhotra et al., 2017). MRI is the most promising of the imaging tools available: it is capable of detecting subtle changes in brain structure and volume in FGR fetuses and infants (Bruno et al., 2017; Polat et al., 2017). There has also been interest in using advanced MRI modalities such as diffusion MRI (dMRI), complemented by specific processing and analysis tools that include diffusion tensor imaging (DTI) and fixel-based analysis (FBA) of constrained spherical deconvolution (CSD) methods, to better delineate and understand the micro-, macro-structural and functional impairments of the FGR fetal and neonatal brain (Eixarch et al., 2012; Arthurs et al., 2017; Pannek et al., 2018). Further, Kannan et al. have performed high fidelity 2D and 3D simulations for predicting and quantifying local and global injuries for organs that include the brain and the lung. These simulations noninvasively “numerically penetrate” the tissues and help reconstruct the optical properties (the presence of water, oxygenated and de-oxygenated blood in tissue), which can predict the extent and severity of organ haemorrhage/injury (Kannan and Przekwas, 2011, Kannan and Przekwas, 2012). However, most of these advanced MRI analysis techniques have not been studied or applied extensively in the newborn brain, and are still in the early development or research stage for their use to detect FGR brain injury (Malhotra et al., 2017). Early and accurate detection of structural and functional brain impairment is not only essential to risk stratify and monitor the affected FGR infant, but also to monitor the response to targeted experimental therapies for FGR, which are increasingly being examined (Nawathe and David, 2018).

In this study, we induced early-onset FGR in fetal sheep (Malhotra et al., 2018) to evaluate the use of advanced MRI brain analysis techniques to detect FGR-related brain injury in the early neonatal period. We then collected the brain for histological analysis to substantiate the presence, distribution and cellular nature of neonatal white matter brain injury in preterm FGR versus appropriately grown newborn lambs.

## 2 Methods

### 2.1 Ethics approval

Experiments complied with the National Health and Medical Research Council (NHMRC) of Australia guidelines for the care and use of animals for scientific purposes and were approved by Monash Medical Centre Animal Ethics Committee A.

### 2.2 Experiment design

Procedures to induce early-onset FGR at mid-gestation and then delivery followed by ventilation at preterm age have been described previously (Alves de Alencar Rocha et al., 2017; Malhotra et al., 2018). In brief, sterile surgery was performed after inducing anaesthesia in twin-bearing Border-Leicester Merino crossbred ewes at 88–90 days gestation (term is 150 days) to induce early onset FGR by single umbilical artery ligation (SUAL) in one of the fetal lambs. The other lamb in the twin pairs was used as the control (appropriately grown for gestational age, AGA).

At 125–127 days gestation, following administration of antenatal steroids in the two days preceding birth, FGR and AGA lambs were delivered and umbilical cord clamped and cut. The lambs were dried, weighed, and transferred to an infant warmer (Fisher and Paykel, Auckland, NZ) where each lamb was intubated (4.0 mm cuffed endotracheal tube), lung liquid passively drained and gentle ventilation commenced. Umbilical venous and artery catheters (internal diameters 5 Fr) were inserted and secured using silk sutures. A pulse oximeter probe (Masimo, Irvine, CA, USA) was placed on the lamb's tail for measurement of transcutaneous oxyhaemoglobin saturation levels (SpO_2_). Ventilation of the preterm FGR and AGA lambs was initiated using assist control ventilation (Babylog 8000+, Dräger, Lüberk, Germany) with an initial peak inspiratory pressure of 30 cm H_2_O and positive end-expiratory pressure of 5 cm H_2_O for the first 10 min and then volume targeted ventilation with tidal volumes of 5–7 ml.kg^−1^ for the remainder of the experiment. The inspired oxygen fraction (FiO_2_) was commenced at 0.3 and then adjusted to maintain lamb SpO_2_ between 85 and 95% after initial resuscitation. All lambs received prophylactic surfactant (100 mg.kg^1^, Curosurf; Chiesi Pharma, Parma, Italy) via the endotracheal tube 10 min after ventilation onset.

As soon as the lamb was stabilised (within 1 h after birth), it was transferred to the MRI scanner (Siemens Skyra 3.0 T, Erlangen, Germany). Lambs were scanned in a supine position and ventilation was maintained using a BabyPac MR compatible ventilator (Pneupac, Smiths Medical, Kent, UK). Throughout ventilation, lambs were sedated by continuous infusion of Alfaxan (Alfaxalone, Jurox, Rutherford, NSW, 3 mg.kg^−1^.min^−1^) through an umbilical venous catheter. The MRI acquisition protocol comprised structural imaging sequences (T1w, T2w), dMRI, and single-voxel proton magnetic resonance spectroscopy (MRS). The total acquisition time was between 60 and 75 min.

At the completion of the experiment, lambs were euthanised by intravenous pentobarbital sodium overdose (100 mg.kg^−1^ I.V.; Valabarb, Jurox, Rutherford, NSW, Australia).

### 2.3 MR Image acquisition

MRI data were acquired using a 15-channel transmit/receive knee coil on the Siemens 3T Skyra (Siemens, Erlangen, Germany) running software version vd13C. Diffusion MRI data were obtained with a single shot echo planar imaging protocol, using the following parameters: coronal slices, repetition time/echo time (TR/TE) = 10,500/99 ms, 1.2 × 1.2 × 1.2 mm^3^ isotropic voxels, 128 × 128 acquisition matrix, GRAPPA = 2 and number of excitations (NEX) = 1. Multi-shell data were acquired with the following shells: 30 directions at b = 500 and 750 s/mm^2^, 64 directions at b = 1000, 1250 and 1500 s/mm^2^, and five volumes without diffusion weighting (b = 0). Additional pairs of b = 0 images were acquired with reverse phase encoding to correct for image distortions. A 3D T2-weighted image was acquired using a fast spin echo sequence with the following parameters: 0.5 × 0.5 × 0.5 mm^3^ isotropic voxels, 384 × 384 acquisition matrix, TR/TE = 1000/130 ms and NEX = 2, Echo Train Length (ETL) = 64. A T1-weighted image was generated using a spin echo sequence with inversion recovery. The parameters were as follow: TR/TE = 1440/3.92 ms, inversion time = 900 ms, NEX = 1, 0.8 × 0.8 × 0.8 mm^3^ isotropic voxels and acquisition matrix = 256 × 256. A 3D SW image was acquired using a 3D FLASH sequence with the following parameters: TR/TE = 28/20 ms, NEX = 2, flip angle = 15°, 0.6 × 0.6 × 0.6 mm^3^ isotropic voxels, and acquisition matrix = 288 × 216.

### 2.4 MRS

**Fig. 1:**
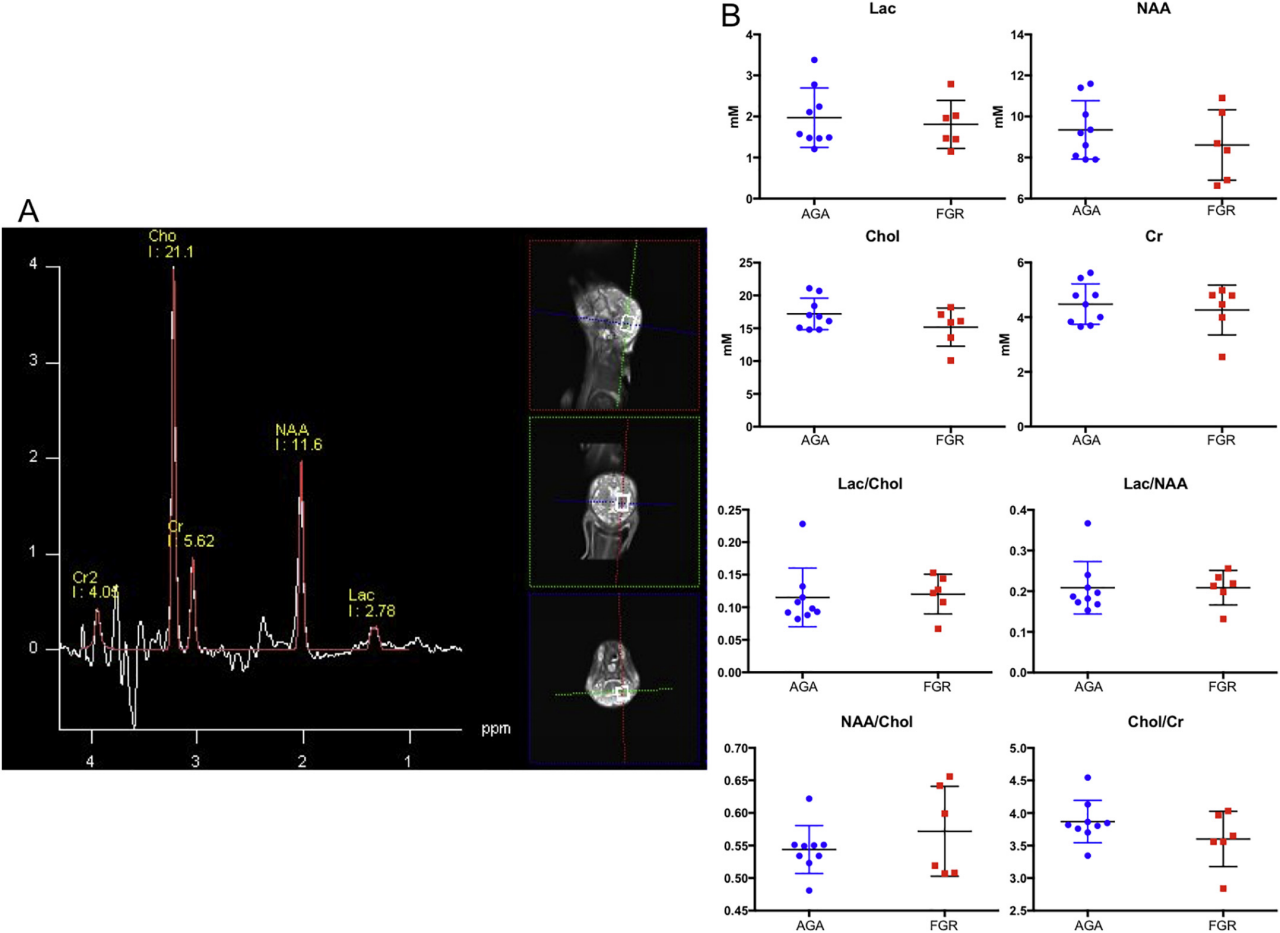
A: Representative screenshot of spectrogram showing the deep brain voxel used to study brain metabolites. B: Graphs showing absolute values and ratios of metabolites in AGA (blue) and FGR (red) lamb brains studied by MRS. Lac - lactate, NAA - *n*-acetylaspartate, Chol - choline, Cr - creatine. (For interpretation of the references to color in this figure legend, the reader is referred to the web version of this article.)

MRS data were acquired using two water suppressed PRESS Spin Echo sequences with TR = 2000, TE = 35 ms and 270, NEX = 128 and 192, respectively. The scans were also repeated with no water suppression. A single 1.4 cm × 1.5 cm × 2 cm voxel in the deep grey matter of the brain (globus pallidus, Fig. 1) was used to calculate absolute metabolite values and metabolite ratios by a using peak area under the MRS curve. Metabolites studied include lactate (Lac), choline (Chol), creatine (Cr), and *n*-acetylaspartate (NAA) and ratios assessed were Lac/choline, Lac/NAA, NAA/choline and choline/creatine.

### 2.5 Diffusion MRI pre-processing

dMRI data were corrected for head motion, eddy currents, and susceptibility distortions using TOPUP and EDDY tools, as implemented in FSL (FMRIB Software Library) (Jenkinson et al., 2012; Andersson and Sotiropoulos, 2016). FSL-BET (Brain Extraction Tool) was used to extract the brain and create a brain mask. The data were denoised using MP-PCA denoising (Veraart et al., 2016) implemented in MRtrix. DTI metrics were calculated from a single shell (b-value = 1500 s/mm^2^) using FSL and included fractional anisotropy (FA), axial diffusivity (AD), radial diffusivity (RD) and mean diffusivity (MD).

### 2.6 Region of Interest voxel-based analysis

**Fig. 2:**
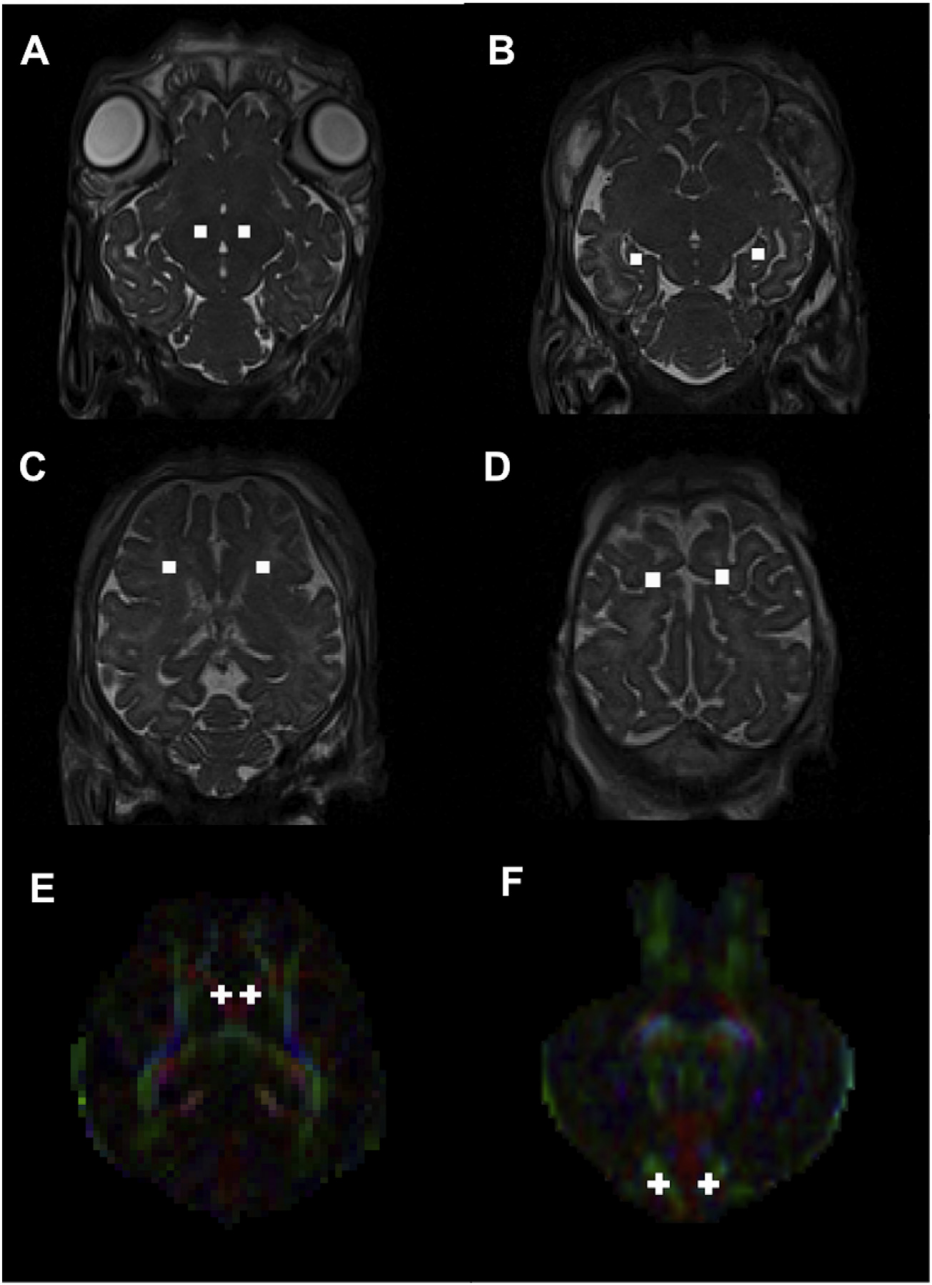
Examples of regions of interest (ROI) from an exemplar lamb brain. A) Thalamus, B) Hippocampus, C) Periventricular white matter and, D) Subcortical white matter regions were first manually identified on T2 images, and then warped to the diffusion space. E) Corpus callosum and, F) cerebellum were accurately visualised and selected on principal diffusion direction maps.

A MATLAB (Version R2017b, The MathWorks, Natick, MA, USA) script was used to automate the generation of spherical regions of interest (ROIs) with identical 3D volumes from a manually determined point on high-resolution T2-weighted brain images. ROIs evaluated included the thalamus, hippocampus, cerebellum, corpus callosum, periventricular white matter (PVWM) and subcortical white matter (Fig. 2). Each ROI was manually inspected to ensure that they covered the same region in all images. T2-weighted and distortion corrected b = 0 images were registered using FSL-FLIRT and the resulting transforms were used to register the ROIs to the dMRI space. The mean and standard deviation of each of the DTI metrics was calculated within all ROIs using MATLAB.

### 2.7 3-Tissue modelling and fixel-based analysis

**Fig. 3:**
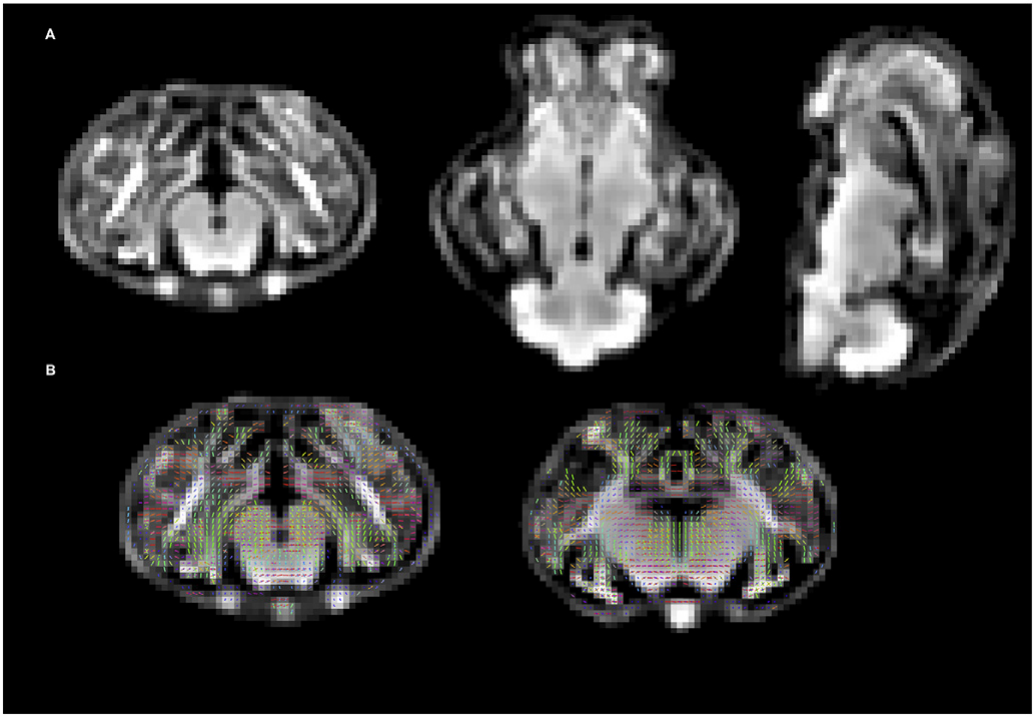
A: White matter FOD templates. Left to right: Coronal, axial and sagittal views. B: White matter template analysis fixel mask, computed from the FOD templates by applying a threshold of 0.08. The white matter fixels are colour-encoded according to orientation: red for left/right; green for anterior/posterior; blue for superior/inferior. (For interpretation of the references to color in this figure legend, the reader is referred to the web version of this article.)

3-tissue response functions for every animal were estimated from the data themselves using an unsupervised method (Dhollander et al., 2016; Dhollander et al., 2019) available in MRtrix3Tissue (https://3Tissue.github.io/), an MRtrix3 fork. The estimated response functions per tissue type were then averaged across all lambs to obtain a unique group response function for each tissue type (white matter-like, grey matter-like and cerebrospinal fluid-like). The dMRI data were up-sampled to an isotropic voxel size of 1 mm and 3-tissue CSD was performed using the multi-shell multi-tissue spherical deconvolution algorithm (Jeurissen et al., 2014), resulting in a white matter Fibre Orientation Distribution (FOD). Global intensity normalisation was performed using multi-tissue informed log-domain intensity normalisation across lambs to correct for global intensity differences between scans. A study-specific population template (Fig. 3) was generated from 5 AGA and 5 FGR lambs by non-linear registration of the white matter FOD images (Raffelt et al., 2011). Equal numbers of AGA and FGR lambs were used to avoid biasing the template to one particular group (Wright et al., 2017). White matter FOD images of all AGA and FGR lamb brain scans were subsequently registered to this population template (Raffelt et al., 2011; Raffelt et al., 2012). A white matter template analysis fixel mask was then computed from the FOD template (Fig. 3).

Fixel-specific fibre density (FD), fibre cross-section (FC), and fibre density and cross-section (FDC) were computed and statistically analysed using connectivity-based fixel enhancement with 5000 permutations, fully corrected for family-wise error (Raffelt et al., 2015; Raffelt et al., 2017). Group comparison was performed between AGA and FGR lambs. The fixel-based analysis was performed using MRtrix3 software (Tournier et al., 2019). It was hypothesised that there might be differences between AGA and FGR lamb brains even without macroscopic evidence of brain abnormality on conventional MRI, and that FGR lambs would show white matter injury compared to AGA lambs, which would be supported by reduction in FC, FD and FDC in the FGR lambs.

### 2.8 Brain pathology

After euthanasia, the brain was removed and weighed. The left brain hemisphere was divided into four sections and frozen for analysis. The right brain hemisphere was cut coronally into 5 mm slices and fixed in formalin for 48–72 h and then embedded in paraffin (ProSci Tech, Thuringowa, Australia) for histological analysis.

### 2.9 Histological staining

Luxol fast blue (LFB) was used to stain lipoproteins present in the myelin sheath of the white matter using a standard protocol in order to detect demyelination under light microscopy (Alves de Alencar Rocha et al., 2017). Hydrated brain paraffin sections were placed in luxol fast blue solution overnight. Excess stain was rinsed off, sections placed in dilute lithium carbonate, differentiated in 70% ethanol, further stained in dilute fuschin and picric acid, and mounted in resinous media. Brain sections were analysed for semi-quantitative assessment of myelin injury and demyelination by a blinded examiner, and scored for presence and quality of luxol fast blue staining, myelin breaks and vacuolation in white matter. Brain sections were examined at the level of the subventricular zone and the paraventricular thalamus (sections 0760 and 1120 respectively, Michigan University Sheep Brain Atlas). Brain sections were thoroughly examined for any signs of demyelination with particular ROIs based on imaging data, including PVWM, corpus callosum, hippocampus and cerebellar white matter.

### 2.10 Statistics

Birth weight, MRS, and ROI-based dMRI metrics are presented as mean and standard deviation (S.D.). Statistical comparisons (*t*-tests between AGA and FGR groups) were conducted using GraphPad Prism (v7, GraphPad Software, San Diego, CA). Significance was accepted when *p* < .05. As mentioned above, fixel-based statistical analysis of grouped data was carried out using the MRtrix3 software (Tournier et al., 2019), also accepting significance when *p* < .05.

## 3 Results

Ten pregnant ewes bearing twin pairs of fetal lambs were surgically instrumented under anaesthesia at 88–90 days lamb gestation to induce FGR in one of the lambs. There were two stillbirths in the FGR cohort, and one early neonatal death in the AGA cohort. Nine AGA and eight FGR lambs completed the MRI brain studies (Table 1). However, two FGR lamb brain scans were excluded due to severe motion artefact and/or failure in acquisition of one or two b-values for dMRI data.

**Table 1:** Demographic characteristics of included lambs.

	AGA	FGR
Lambs – live born	12/12	9/12
Lambs - MRI analysis	9/12	6/9
Sex, male: female	4:5	3:3
Birth weight (kg), mean (SD)	3.3 (0.3)	2.2 (0.5)⁎
Birth order, first: second	6:3	2:4

⁎ Denotes significant difference (*p* = .002, unpaired *t*-test).

At birth, the body weight of the FGR lambs was significantly reduced compared to the AGA lambs, 2.2 ± 0.5 kg vs. 3.3 ± 0.3 kg, respectively (t-test, *p* = .002). Lambs were scanned in the 3T MRI scanner in the order they were born (AGA: 6 first born, FGR: 2 first born). Lamb scanning was completed within a median of 3 (2.5–4) hours after birth.

**Fig. 4:**
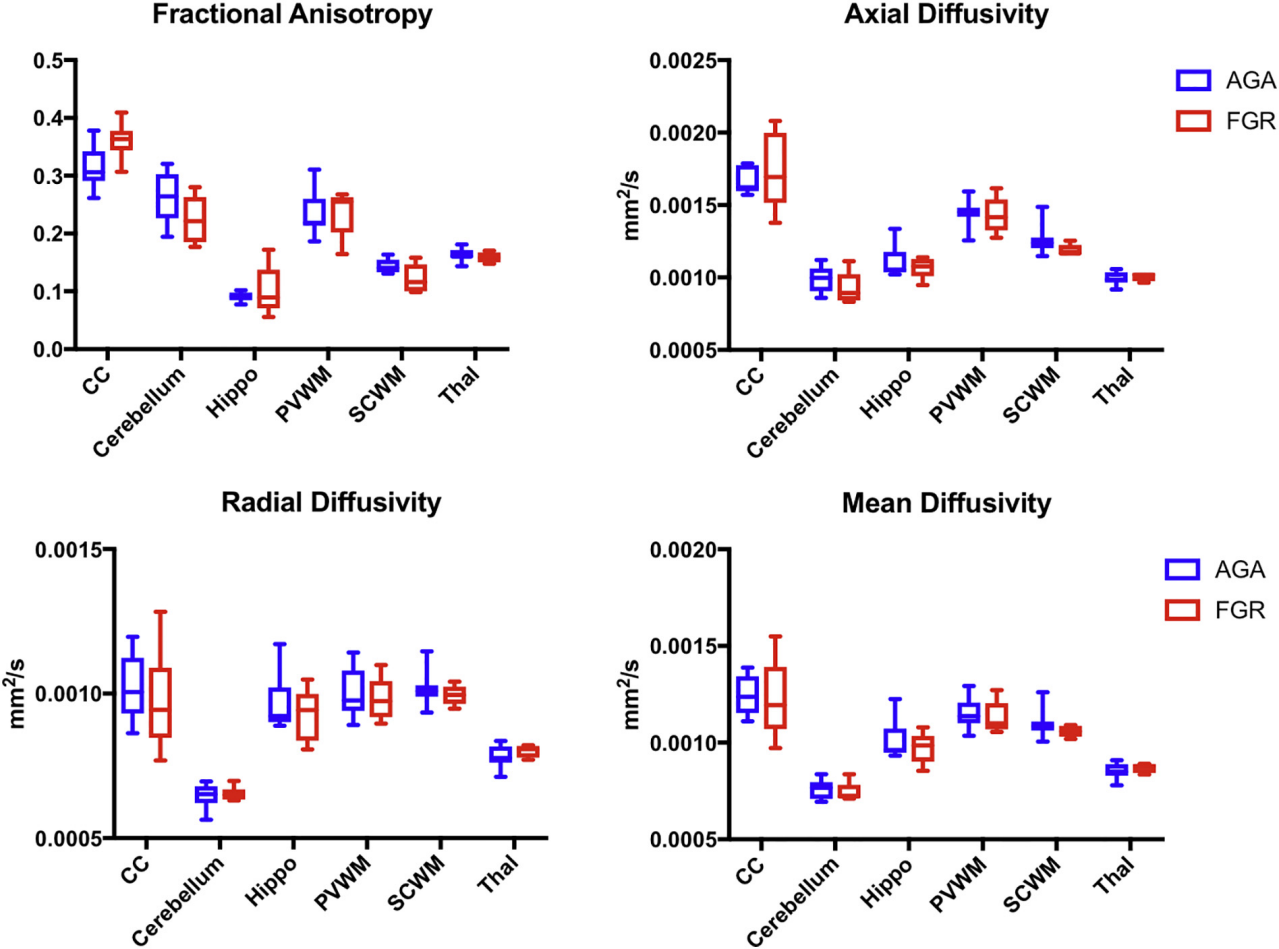
DTI characteristics of ROIs calculated from ROI-based analysis. Thal - thalamus, Hippo - hippocampus, PVWM - periventricular white matter, SCWM - subcortical white matter, CC - corpus callosum.

Assessment of structural brain scans (T1w, susceptibility weighted imaging) of all lambs was conducted by a paediatric radiologist (MD) blinded to the groups. There were no structural brain lesions identified in any scans. MRS metabolite absolute values and metabolite ratios are shown in Fig. 1. There were no statistically significant differences found in any of the metabolites or ratios between the lamb groups. ROI-based analysis of dMRI data generated mean FA, AD, MD and RD values for each region of interest, and these were plotted for both lamb groups (Fig. 4). There were no statistically significant differences seen for any DTI metric in any of the ROIs studied.

**Fig. 5:**
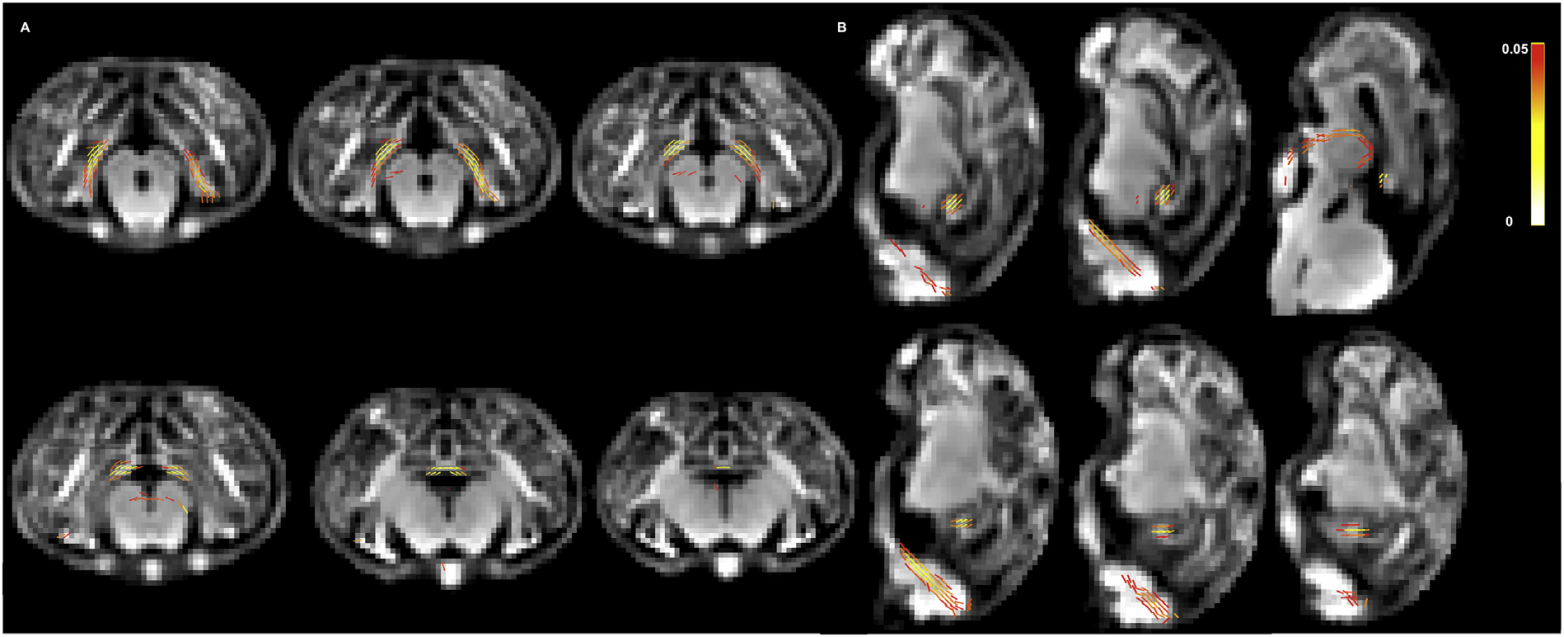
A: Fixel-based analysis of 3-tissue constrained spherical deconvolution of dMRI data (coronal view). Specific tracts in hippocampus, thalamus, periventricular white matter and cerebellum showed a decrease in fibre content (FC) in FGR vs. AGA lamb brains (seen as coloured tracts in family-wise error corrected fixel mask). B: Fixel-based analysis of 3-tissue constrained spherical deconvolution of dMRI data (Sagittal view). Specific tracts in hippocampus, periventricular white matter, thalamus, and cerebellum show a significant decrease in fibre content (FC) in FGR vs. AGA lamb brains (seen as coloured tracts in family-wise error corrected fixel mask).

**Fig. 6:**
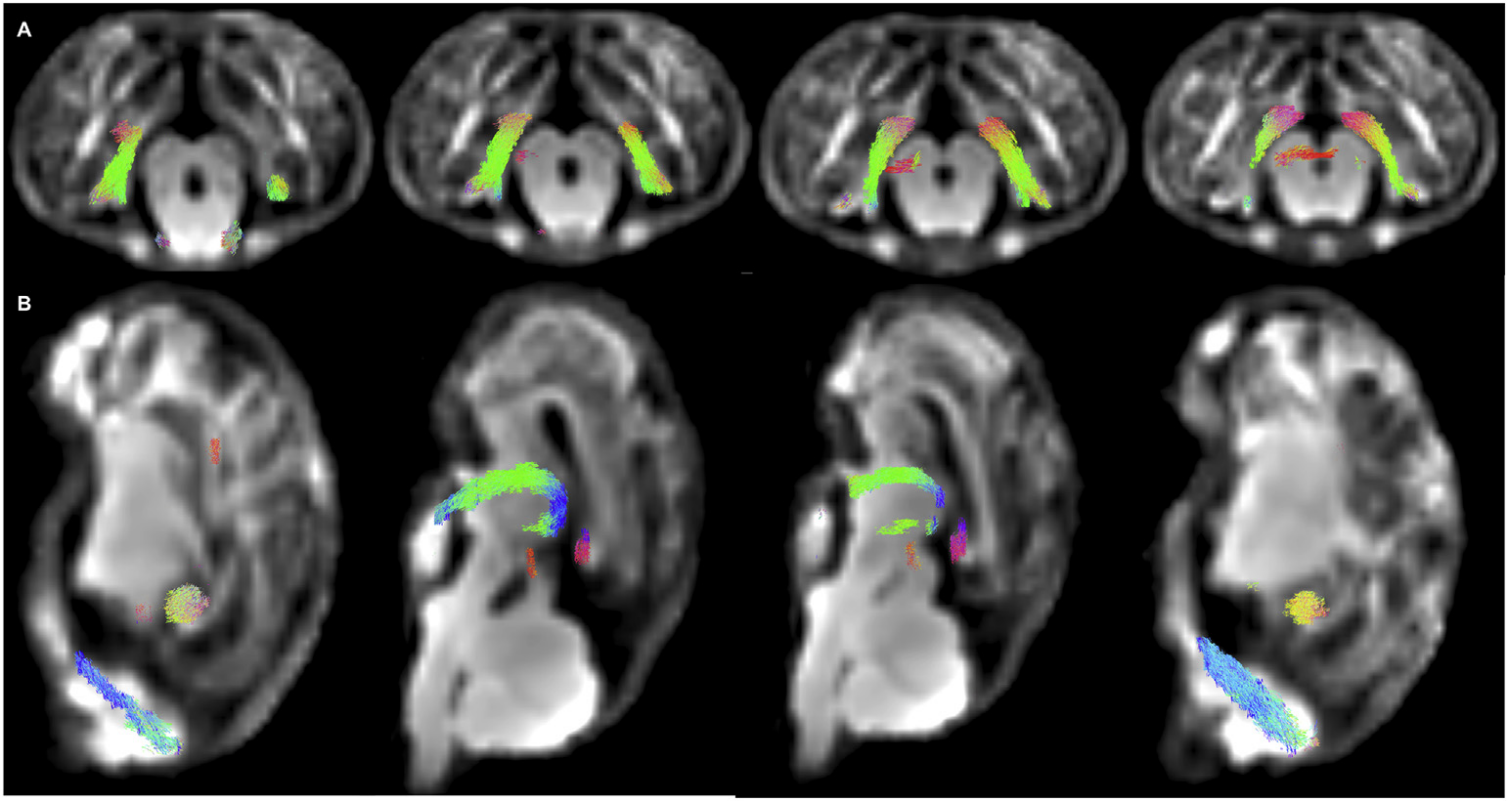
A: Coronal views of significant fixel based analysis results in log FC as streamlines. Colours represent the direction of the tracts. Red for left/right; green for anterior/posterior; blue for superior/inferior. B: Sagittal view of significant results in FC as streamlines. Colours represent the direction of the tracts. Red for left/right; green for anterior/posterior; blue for superior/inferior. (For interpretation of the references to color in this figure legend, the reader is referred to the web version of this article.)

Fig. 5A and B illustrate the results of the group comparison between AGA and FGR group using the fixel-based analysis of dMRI data for coronal and sagittal views, respectively. The results are overlaid on the fixel template and highlight the fibres with statistically significant (*p* < .05) differences. Fig. 6 visualises these results using streamlines, specifically for the fibre cross-section metric. FC was significantly reduced in FGR lambs compared to AGA within the PVWM, hippocampus, and cerebellar white matter regions of lamb brains. The changes were observed in bilateral hemispheres of the lamb brains (Fig. 6). There were no statistically significant differences observed between the groups for the other fixel measures, FD and FDC.

**Fig. 7:**
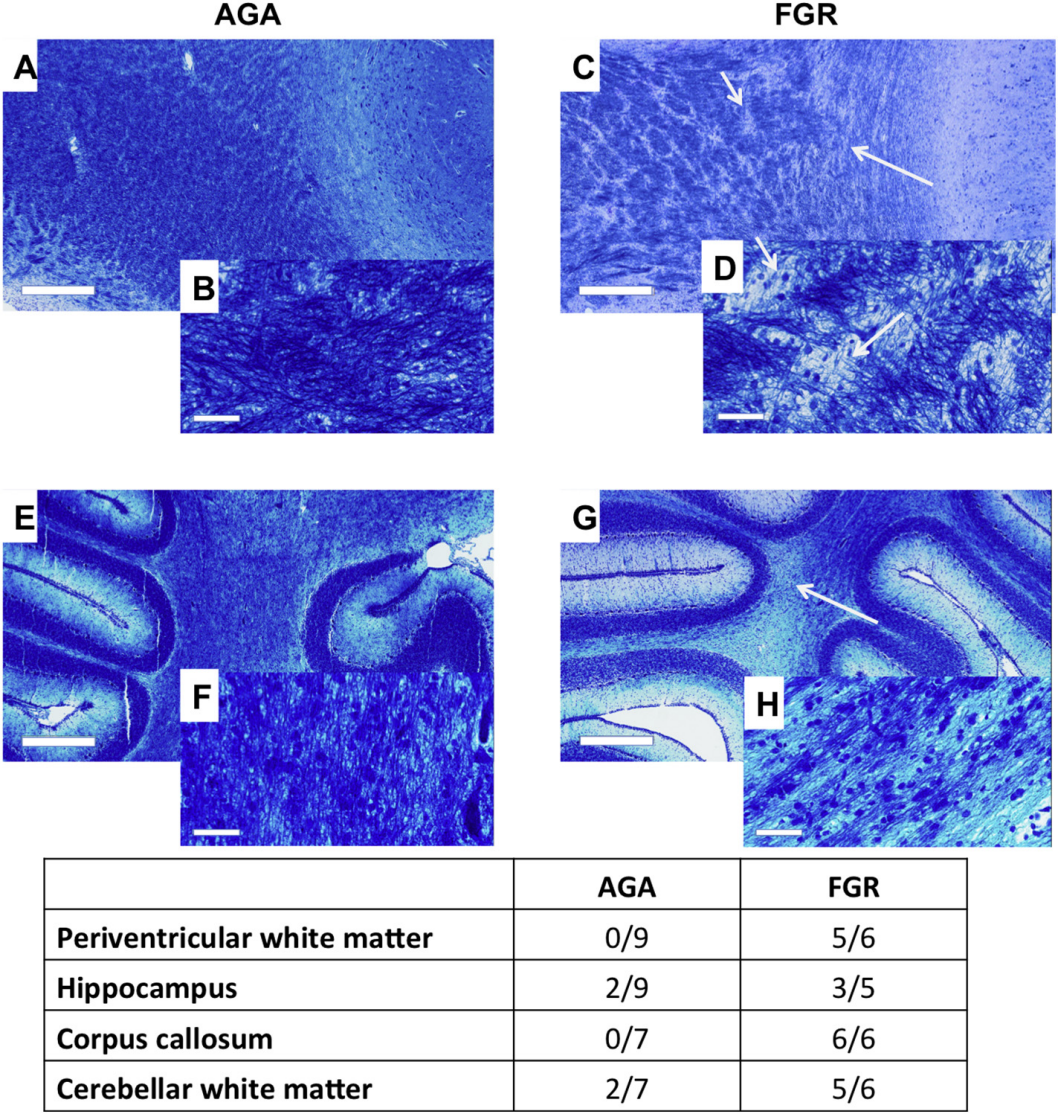
Representative photomicrographs (low and high magnification) of luxol fast blue (LFB) staining of regions of interest in AGA and FGR lamb brains. A-B: Periventricular white matter in AGA lambs showing uniform LFB staining. C-D: Periventricular white matter in FGR lambs showing patchiness in LFB staining (arrows). E-F: Cerebellar white matter in AGA lambs. G-H: Cerebellar white matter in FGR lambs showing breaks in the myelin, thinning of myelin tract and areas devoid of LFB stained myelin (arrow) (H). Scale bars: large: 500 μm, small: 50 μm.

Luxol fast blue was used to assess abnormalities in myelin organisation and evidence of demyelination. Luxol fast blue staining showed substantial hypomyelination in the white matter of FGR lambs compared with AGA lambs, most notable in the corpus callosum, the subcortical and PVWM, the subcallosal bundle, external capsule, the cingulum bundle and white matter tracts of the hippocampus and cerebellum. Myelin disruptions were evident as the presence of patchiness in the staining of myelin tracts, and demyelination as an overall reduction in the density of luxol fast blue staining in the FGR group compared to the AGA group. Within cerebellar white matter, FGR lamb brains showed myelin breaks and a decrease in luxol fast blue staining, thinning of the white matter tracts in the cerebellar lobes, and the presence of vacuoles, throughout the white matter (Fig. 7). In the PVWM and corpus callosum, irregularities and loss of luxol fast blue staining were seen in FGR lamb brains compared with AGA brains. The white matter abnormalities observed in these brain regions were seen as areas completely devoid of staining (Fig. 7). In the hippocampus, myelin irregularities were detected in the fornix, alveus, angular bundle and fimbrial regions of some FGR lamb brains. The relative numbers of brain regions of FGR and AGA lambs that showed evidence of abnormalities in the white matter are presented in the table (Fig. 7).

## 4 Discussion

In the current study we used advanced MRI brain imaging techniques for the identification of FGR related brain injury in newborn lambs and, for the first time, the presence of FGR related brain injury observed on imaging was confirmed on histological staining. No differences in brain structure and metabolite levels were observed on conventional MRI sequences and MRS between FGR and AGA lambs. However, advanced fixel-based analysis revealed macrostructural changes in the form of reduced FC in the PVWM, hippocampus, and cerebellar tracts of the FGR lamb brain, which was confirmed on histological staining of tracts in those regions.

The early detection and assessment of perinatal brain injury is critical in conditions that put the fetal or neonatal brain at risk, such as in FGR (Malhotra et al., 2017). The neuropathology associated with placental insufficiency and FGR is often subtle, and therefore not always apparent on conventional imaging, particularly in the neonatal period (Malhotra et al., 2015; Malhotra et al., 2017). Studies of the newborn FGR brain using MRI and MRS studies have shown variable results. Some studies have shown decreased total volume and/or specific regional volume changes when compared to AGA infants (Tolsa et al., 2004; Dubois et al., 2008), while others show no significant differences (Ramenghi et al., 2011). In some studies, the differences in brain structure become more apparent on MRI scans later in infancy (Padilla et al., 2011; Padilla et al., 2014). Brain metabolite concentrations were altered in a preclinical study in newborn FGR rabbits, with lower levels of aspartate and *n*-acetylaspartate in cortical and hippocampal regions, and increased glycine in the striatum (Simoes et al., 2015). MRS undertaken during pregnancy shows that growth restricted fetuses with brain sparing have lower NAA:Cr and NAA:Cho ratios compared to AGA fetuses, indicative of reduced neuronal number and/or function (Story et al., 2011). In contrast, a study on small for gestational age infants studied at two time points early in the neonatal period found no differences in cerebral metabolism (Roelants-van Rijn et al., 2004). Similarly in our lamb study, we did not observe differences using a single voxel estimation of deep grey matter metabolism in FGR and AGA lamb brains. This result suggests that, at least in deep grey matter encompassing basal ganglia, there is no loss of neuronal number or function within the FGR brain. It is of course possible other brain regions might have a different metabolite profile. It is also possible that the chronic but stable nature of antenatal compromise induced via placental insufficiency in this lamb model precluded any changes in brain metabolite concentrations as seen on MRS.

There is very limited literature on DTI based analysis in the FGR newborn brain. A study in FGR (small for gestational age infants) and AGA neonates showed lower FA and higher diffusivity (AD, MD, RD) in FGR infants as compared to AGA infants in a number of brain regions out of a total of 122 brain regions studied (Wang et al., 2017). Similarly, a study in FGR rabbits also showed altered brain networks and decreased FA in the hippocampus (Illa et al., 2018). We did not observe any changes in DTI measures in the six brain regions studied within the FGR lamb brain. DTI metrics are voxel-specific, not fibre-specific and hence, it is possible that the voxels included white matter regions with crossing fibres that have differing DTI characteristics, hence masking the effects of a disorganised white matter structure (Jeurissen et al., 2013; Wright et al., 2017). It is also possible that specific brain regions may have differing characteristics within a diseased brain or disease model. We used a ROI specific model for the DTI analyses while the FBA analyses studied the whole white matter region in the template. This may also account for the lack of DTI changes despite significant pathology seen on histological staining.

FBA was used to study fibre specific changes in the brain. This technique enables detection of the morphological alterations in axonal tracts that may or may not contribute to differences in voxel-based morphometry (Raffelt et al., 2017). FBA allows the assessment of tissue microstructure (FD), local macrostructure (FC), and the combined effect of both microstructure and macrostructure (FDC). Disease-associated axonal loss typically leads to reduced FD (Raffelt et al., 2015), while a reduced FC generally represents axonal loss associated with the extra-axonal space filled with extracellular matrix and cells, related to inflammation or gliosis (Raffelt et al., 2012). Reduced FC may also result from long term atrophy of the fibre bundle (as seen in Alzheimer's or motor neuron disease) but in most cases neurodegenerative diseases lead to reduction in FDC, the combined measure (Raffelt et al., 2017). Pannek et al. recently used fixel-based analysis to study the micro- and macrostructural differences in brain scans of preterm- and term-born infants at term equivalent age (Pannek et al., 2018), and found that this analysis technique was useful for detection of both microstructural and macrostructural abnormalities in infants born preterm. In the current preclinical study, we observed differences in fixel-based analysis results in specific white matter regions of the FGR lamb brains compared to animals that were appropriately grown. In particular, differences in FC were noted between FGR and AGA lambs in the PVWM, hippocampus and cerebellar tracts of the lamb brains. A decrease in FC may signify altered tissue macrostructure, possibly as a consequence of decreased myelination (Gajamange et al., 2018). This was confirmed on histological staining for the first time, which is important, as the changes we see on FBA may signify subtle structural changes previously not appreciated on MRI. The changes in white matter tracts of the FGR brain on FBA, coupled with evidence of hypomyelination and disorganisation on neuropathology, confirm the vulnerability of white matter development in an environment of chronic antenatal hypoxia associated with FGR. That we could detect macrostructural white matter deficits immediately after (preterm) birth confirms that brain injury occurs antenatally. We have previously shown axonal changes and axonal injury in brain white matter regions in both fetal and neonatal studies of FGR sheep (Miller et al., 2014; Alves de Alencar Rocha et al., 2017; Malhotra et al., 2018), which may be associated with the FBA changes seen here. In turn, neonatal white matter deficits and altered brain connectivity may underlie neurodevelopmental disorders that become evident in childhood (Miller et al., 2016).

The animal model lends us the distinct advantage of being able to study brain histological changes that have been identified as areas of interest using brain imaging. We have reported previously that white matter hypomyelination and disorganisation are principal neuropathologies present in FGR lambs (Miller et al., 2014; Alves de Alencar Rocha et al., 2017), but the current study extends the preclinical utility of this work into a clinical understanding of how early detection of FGR brain injury may be improved, and its histological basis. Further work on how other neuropathologies in high risk perinatal conditions may correlate with FBA should to be undertaken to ascertain whether this technique will also be useful to detect other subtle brain changes (Rana et al., 2019). It is also interesting that while we could observe demyelination and disorganisation on histological assessment, this did not result in significant alteration in FD or FDC, or indeed other DTI metrics, which also highlights the need for further studies to specifically correlate imaging with histological analysis. It is not known what degree or nature of white matter injury results in functional deficits, and therefore further preclinical lamb studies should combine brain imaging with functional and histological assessments to obtain a global picture of the relationships between these outcomes. Therefore, future FBA studies should include correlating FBA changes with neurodevelopment in childhood, and whether antenatal or postnatal therapies modify macrostructural and microstructural white matter development. An advantage of undertaking this study in lambs is that we could deliver all animals preterm, at an age equivalent to moderate to late-preterm human brain development (≥32 weeks human gestation) (Alves de Alencar Rocha et al., 2017). In doing so, we were able to detect macrostructural deficits within the FGR brain. We do however note that delivering all lambs preterm is also a study limitation, because we cannot then compare with a term-born group to further characterise potential differences between preterm and term birth, as shown by Pannek recently in a human cohort (Pannek et al., 2018).

## 5 Conclusions

Advanced MRI brain analysis techniques offer novel modalities to image and study perinatal brain injury. In this preclinical study of placental insufficiency and subsequent FGR, we found significant differences in fibre bundle cross-section, indicative of reduced tissue macrostructure within white matter regions. Histological analysis of the same white matter regions showed that these FC changes could be attributed to hypomyelination and disorganisation of axonal tracts. The introduction and use of fixel-based analysis to study perinatal brain structure and function may be an important addition for the comprehensive assessment of brain injury in high-risk populations, including growth restricted infants.
